# Clinical evaluation of lingua plicata in adults

**DOI:** 10.11604/pamj.2025.50.5.45838

**Published:** 2025-01-02

**Authors:** Devesh Nagpure, Sheetal Asutkar

**Affiliations:** 1 Department of Shalyatantra, Mahatma Gandhi Ayurved College Hospital and Research Centre, Salod (H), Datta Meghe Institute of Higher Education and Research (Deemed to be University), Wardha, Maharashtra, India

**Keywords:** Oral mucosa, genetic predisposition, oral Irritation, systemic disorders

## Image in medicine

A 35-year-old female patient visited the Shalyatantra outpatient department, with a three-month history of tongue discomfort and surface fissuring. Clinical examination revealed deep grooves and fissures on the dorsal surface of the tongue, consistent with fissure tongue (lingua plicata). This benign condition has no established etiology but may be associated with genetic factors, local influences, or systemic disorders. The patient's medical and family history were unremarkable, and she exhibited no signs of associated systemic conditions such as Sjögren's syndrome or Melkersson-Rosenthal syndrome. While the fissures were generally asymptomatic, the patient reported occasional mild irritation, particularly upon consumption of spicy foods. Differential diagnoses for fissure tongue included geographic tongue, which typically presents with migratory, smooth, erythematous areas bordered by raised edges that change location, and oral lichen planus, characterized by white striations or reticular patterns on the oral mucosa. Another condition to be considered is median rhomboid glossitis, which manifests as a symmetrical erythematous region on the dorsum of the tongue. The diagnosis of fissure tongue was confirmed based on the clinical presentation and the exclusion of other potential disorders. Management of fissure tongue is primarily supportive and focuses on maintaining proper oral hygiene and avoiding irritants. In this case, no surgical or pharmacological intervention was indicated. The patient was informed about the benign nature of her condition and was educated on oral hygiene practices to minimize potential irritation and prevent debris accumulation within the fissures.

**Figure 1:**
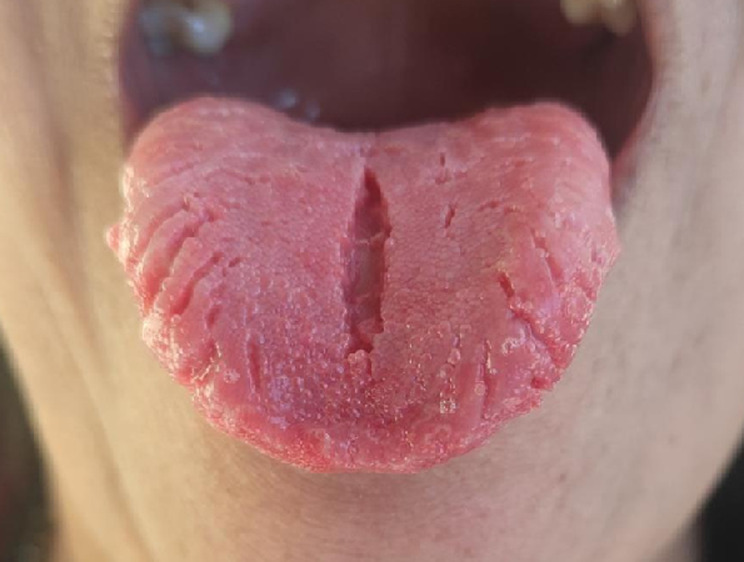
fissured tongue showing prominent median fissure and multiple smaller lateral fissures on the dorsal surface of the tongue

